# How wind drives the correlation between leaf shape and mechanical properties

**DOI:** 10.1038/s41598-018-34588-0

**Published:** 2018-11-05

**Authors:** Jean-François Louf, Logan Nelson, Hosung Kang, Pierre Ntoh Song, Tim Zehnbauer, Sunghwan Jung

**Affiliations:** 1 Department of Biomedical Engineering and Mechanics, Virginia Tech, Blacksburg, VA 24061 USA; 2 Department of Mechanical Engineering, Aix-Marseille Universite, Marseille, France; 3 Department of Biological and Environmental Engineering, Cornell University, Ithaca, NY 14853 USA

## Abstract

From a geometrical point of view, a non-sessile leaf is composed of two parts: a large flat plate called the lamina, and a long beam called the petiole which connects the lamina to the branch/stem. While wind is exerting force (e.g. drag) on the lamina, the petiole undergoes twisting and bending motions. To survive in harsh abiotic conditions, leaves may have evolved to form in different shapes, resulting from a coupling between the lamina geometry and the petiole mechanical properties. In this study, we measure the shape of laminae from 120 simple leaf species (no leaflets). Leaves of the same species are found to be geometrically similar regardless of their size. From tensile/torsional tests, we characterize the bending rigidity (*EI*) and the twisting rigidity (*GJ*) of 15 petioles of 4 species in the Spring/Summer: Red Oak (*Quercus Rubra*), American Sycamore (*Platanus occidentalis*), Yellow Poplar (*Liriodendron tulipifera*), and Sugar Maple (*Acer saccharum*). A twist-to-bend ratio *EI*/*GJ* is found to be around 4.3, within the range in previous studies conducted on similar species (*EI*/*GJ* = 2.7~8.0 reported in S. Vogel, 1992). In addition, we develop a simple energetic model to find a relation between geometrical shapes and mechanical properties (*EI*/*GJ* = 2*L*_*L*_/*W*_*C*_ where *L*_*L*_ is the laminar length and *W*_*C*_ is the laminar width), verified with experimental data. Lastly, we discuss leaf’s ability to reduce stress at the stem-petiole junction by choosing certain geometry, and also present exploratory results on the effect that seasons have on the Young’s and twisting moduli.

## Introduction

Photosynthesis is the principal mechanism for nutrition in plants. Although there are several photosynthetic pathways for different species^1^, the fundamental step is the same: using light energy to transform water and CO_2_ into sugar and oxygen^2^. In trees, leaves have evolved to perform the photosynthesis function, and typical non-sessile leaves are composed of a petiole and a lamina. The petiole is a beam-like structure connecting the lamina to the stem, while the lamina is the major photosynthetic part in leaves. The lamina appears to be green and flattened in a plane perpendicular to the stem, which is presumably configured to maximize the capture of sunlight^3^.

In nature, trees have evolved to have many different leaf forms in terms of size, lobes, and orientation^4^. But from a simple mechanics perspective, one can expect that the most optimized leaf would have a large, flat, and stiff lamina to maximize the light capture, and a flexible petiole to avoid fracture^5,6^. However, the ability to deploy leaves to sunlight^7–9^ regardless of external factors, such as wind^10,11^ is crucial in plant survival^12^. Combined with other internal factors, such as optimal sap flow^13^, mechanics may have led angiosperm leaves to today’s large diversity in shape.

Plants are able to produce only a limited amount of biomass over time^14^. As a result, plants optimize the biomass allocation to assure both growth and survival^15,16^. For example, if a stem is transiently bent the stem stops its longitudinal growth and allocates its biomass to strengthen itself. This results in a larger diameter and bigger roots to have a better resistance to bending and a better anchorage on the ground^17–21^. If we apply a similar reasoning to leaves, we can think of a trade off between the lamina shape and the petiole mechanical properties^22,23^. The petiole has to furnish the best mechanical support for the lamina to stably photosynthesize. This may result in a coupling between the lamina shape and the petiole’s mechanical properties.

While other studies have focused on large leaves^24,25^ where self-support appears to be the principal criterion for evolution^26^, our study targets more common leaves of length on the order of ten centimeters. In this work, we characterize the morphology of 114 leaves^27^ and also conduct quantitative measurements on 15 leaf samples from four different species (Red Oak (*Quercus Rubra*), American Sycamore (*Platanus occidentalis*), Yellow Poplar (*Liriodendron tulipifera*), and Sugar Maple (*Acer saccharum*)) in the lab. In particular, we measure accurate geometrical properties (lamina shapes and petiole cross-sections) as well as mechanical properties (Young’s modulus and shear modulus) for these four species. The underlying idea is to develop a functional relationship between lamina shapes and petiole mechanical properties to provide a better understanding of a leaf’s ability to cope with different stresses (mainly bending and twisting). We also introduce a simple energetic model that quantitatively compares and predicts the bending and twisting of a leaf due to wind drag. Finally, we find the correlation between lamina shapes and petiole mechanical properties.

## Material and Methods

### Leaf samples

Leaves were collected in spring/summer from four tree species: Red Oak (*Quercus Rubra*), American Sycamore (*Platanus occidentalis*), Yellow Poplar (*Liriodendron tulipifera*), and Sugar Maple (*Acer saccharum*), found in the gardens of Virginia Tech, USA. Soon after the branches were cut, they were transferred to the lab and supplied with water within a few minutes. Pictures of leaf samples were taken, and then mechanical tests on the samples were conducted (see the details in later sections). Shortly after the last mechanical test, the leaves were put in wet paper towels in closed zip bags. Each petiole sample was scanned by a high-resolution *μ*CT scan (Bruker, Skyscaner1172) with the X-ray source power of 50 *kV*, 150 *μA*. The CT-scan images were reconstructed using a NRecon software to obtain cross-sections of each sample. For detailed CT scans, several leaves are designated to be scanned without mechanical tests. All tests on each sample were done within a few hours after the branches were cut.

### Characterizing Leaf Morphology

To investigate the morphology of leaves, geometrical measurements were conducted on petioles and laminae. We focused on leaves from 4 species with lobed bases and long petioles, as they experience less drag and flutter less than leaves with acute bases and short petioles^28^.

#### Lamina/petiole morphology

**Figure 1:**
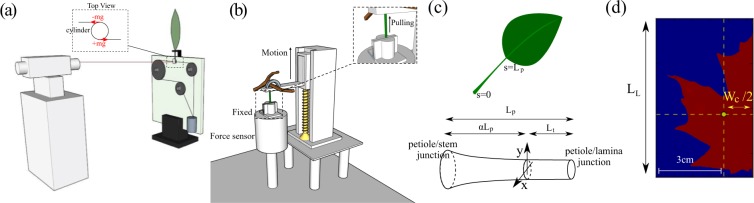
(**a**) Schematic of twisting test apparatus. One end of a petiole is fixed, while the other end is connected to a small cylinder. Two wires, one glued on the front of the cylinder and one on its back (see the inset), are connected via the use of pulleys to a platform where masses can be added. When we add masses, torsion is applied on the petiole. To measure the torsion, we use a mirror and a laser. The mirror is glued to the front of the cylinder, and the laser is set in front of the same cylinder in order to be reflected by the mirror. When torsion is applied, the mirror reflects the laser with a small angle that can be measured using the screen located behind the laser. When looking at the petiole, we observed the torsion located between the petiole/lamina end and the middle of the petiole. (**b**) Pull-off apparatus. The end of the petiole located close to the stem is connected to a linear motor that allows only vertical displacement. The other part of the petiole is linked to a force sensor. A tensile test is done until rupture. The rupture always happens at the junction of the petiole and the stem. (**c**) Schematic of a leaf above a schematic of a petiole. The arclength coordinate *s* is measured along the petiole, equaling 0 close to the stem, and L_*p*_, the length of the petiole, close to the lamina. On the schematic of the petiole we can see the length L_*t*_, which is the characteristic length where twisting occurs. (**d**) Image of the left half of a Maple leaf. The yellow point indicates the location of the centroid. The same image analysis is performed on the right part of the leaf, and the distance between the two centroids is defined as *W*_*c*_.

Photos of leaves were taken to measure the lamina length *L*_*L*_, lamina width *W*_*C*_, and petiole length *L*_*P*_. The lengths, *L*_*L*_ and *L*_*P*_, are directly measured from the photos with a ruler for reference. For the width measurement, we used PYTHON’s image toolbox in addition with a customized code to find the centroid position of each half of laminae. The distance between two centroids *W*_*C*_ is then calculated as shown in Fig. 1(d).

#### Petiole cross-section morphology

**Figure 2:**
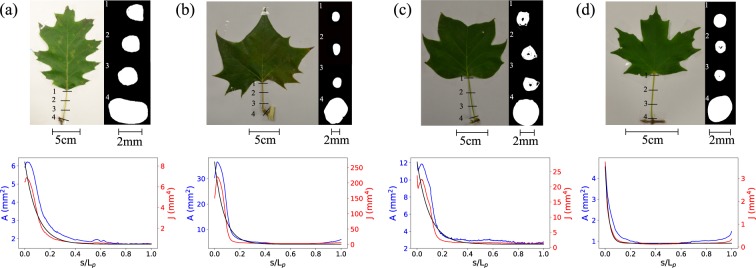
Pictures of the leaves of the four species studied, from left to right: (**a**) Red Oak, (**b**) American Sycamore, (**c**) Yellow Poplar, and (**d**) Sugar Maple. Under each picture there is a plot of the area *A* and the polar moment of Inertia *J* along the petiole. An exponential fit done on J (in black) allows us to calculate *α* × *L*_*p*_ corresponding at the location where *J* is decreased by 99%. This coefficient *α* is then used to accurately determine the twisting length *L*_*t*_ = (1 − *α*)*L*_*p*_.

The exact shape of the petiole cross section has been precisely measured using a *μ*CT-scan as described in section 2.1. We observed that the cross-sectional area, *A*, at the petiole/stem junction is always bigger than the one at the petiole/lamina junction (Fig. 2). Then, we measured the second moments of area *I*_*X*_ and *I*_*Y*_; defined as ${I}_{X}=\int {y}^{2}dydx$, ${I}_{Y}=\int {x}^{2}dxdy$, under the assumption of an isotropic and homogeneous material where *x* and *y* are horizontal and vertical coordinates from the centroid. The polar moment of area, *J*, is defined as the sum of the two second moments of area *I*_*X*_ and *I*_*Y*_. We were thus able to obtain *I*_*X*_, *I*_*Y*_, and *J* everywhere along the petiole. All values calculated using the *μ*CT-scan are an average of 50 representative images (1.1–1.4 mm along the axial direction).

### Mechanical Properties

To understand the twisting and bending motions, torsional and tensile tests have been conducted on petioles.

#### Twisting length

Niklas *et al*.^29^ have shown that there are two types of petioles: one with uniform cross-sectional area and stiffness (*EI*), and one which is tapered with a basipetal increase of the stiffness. For the latter case, twisting does not occur uniformly along the petiole, but rather develops more in the portion of smaller second moment of area. As a result, in our twisting experiment, to accurately measure *GJ* we need to know what the characteristic twisting length is. With measured *A* and *J*, we fit *J* with an exponential function and calculate the 99^*th*^ percentile of its maximum value (Fig. 2). Then, the distance from this point to the lamina is defined as our twisting length *L*_*t*_ (Fig. 1(c)), which will be used in the calculation of *GJ*.

#### Torsional test

A new experimental set-up inspired by Vogel^30^ has been designed (Fig. 1(a)), which allows us to precisely measure the global torsional rigidity *GJ*. The petiole/lamina junction was fixed on an upper holder, and the other end (petiole/stem junction) was clamped on a lower cylinder. On the front and back sides of the cylinder, two wires were attached and linked to pulleys, which are fixed onto a platform with a weight. By changing the weight, we were able to control the torsional force applied to the petiole. To measure this torsional angle, a mirror was attached on the cylinder with a laser pointed at it. When torsion is applied, the cylinder rotates and then reflects the laser light in a different direction. Using this method, we can translate and amplify the small rotational motion to the large linear displacement of the laser on a white screen. By measuring the distance between the initial and final positions of the laser on the screen, we were able to back-calculate the deflection angle, and thereby the rotational deformation. The accuracy of our set-up was confirmed by conducting a few test experiments on cylindrical bars of elastomer (Zhermack Double Elite 8, and 22), whose Young’s moduli were measured using an Instron machine (*E* = 0.2 and 0.9 MPa, respectively). Assuming that the polymer has a Poisson’s ratio of 1/2, we obtain the following relation between the twisting and Young moduli: *G* = *E*/3. We found the difference between the experimental tests and the expected values to be less than 5%.

#### Tensile test

The next mechanical test is an experiment in which leaves are pulled as shown in Fig. 1(b). The tensile tests were conducted on petioles in order to measure the traction Young’s modulus. The lamina end of the petiole was squeezed and fixed on a force sensor (LCM105-10; Omegadyne, Inc.), while the stem end was fixed to a linear stage (see the inset of Fig. 1(b)). A constant pulling speed of 1.27 mm/s was applied, and the force acting on the petiole was recorded from the sensor. Tests were perfomed to failure of the petiole, which always occurred at the petiole/stem junction. The stress was calculated after measuring the petiole cross section at the junction where it failed, and the strain was measured from the displacement of the linear stage. Finally, we plotted a stress-strain curve, giving us the Young’s modulus *E* of the petiole.

## Results

When subjected to wind, a long slim lamina will induce more bending stress to its petiole than shearing stress. In contrast, a short wide lamina will produce more shearing stress associated with twisting motions than bending stress to its petiole. In order to be compliant with either twisting or bending forces resulting from its lamina shape, the petiole presumably has an optimized shape and mechanical properties. In this study, we characterized the Young’s modulus *E* (Pa), the shear modulus *G* (Pa), the second moment of area *I*_*X*_ (m^4^), and the polar moment of area *J* (m^4^), and checked for correlations with lamina shape.

### Flexural stiffness ratio

Using data obtained from the morphology measurements and mechanical tests, we were able to access the flexural rigidity *EI*_*X*_. The variable *I*_*X*_ is calculated in the circular part of the petiole (local quantity). The flexural rigidity quantifies the resistance of a leaf to pull-off after bending, and the twisting rigidity quantifies its resistance to twist-off. These two quantities can be used to calculate the flexural stiffness ratio *EI*_*X*_/*GJ* defined by Vogel^30^. Moreover, by writing a simple force balance between the twisting and bending of a leaf resulting from the action of the wind, we can also write a more accurate scaling law.

The drag *M*_*drag*_ (N · m) exerted by wind on a lamina scales as

1 $${M}_{drag}\approx \frac{1}{2}\rho {U}^{2}{L}_{L}{W}_{C}\frac{{L}_{L}}{2}$$

where *ρ* (kg/m^3^) is the air density, and *U* (m/s) the wind speed. Drag on a lamina exerts a bending moment on the petiole, which gives rise to a bending angle Θ^*bend*^ as

2 $${{\rm{\Theta }}}^{bend}=\frac{{M}_{drag}{L}_{P}}{E{I}_{X}}\mathrm{.}$$

Similarly, we can write torque *T*_*drag*_ (N · m) resulting from the drag as

3 $${T}_{drag}\approx \frac{1}{2}\rho {U}^{2}{L}_{L}\frac{{W}_{C}}{2}\frac{{W}_{C}}{2},$$

and the associated twisting angle Θ^*twist*^ is given as

4 $${{\rm{\Theta }}}^{twist}=\frac{{T}_{drag}{L}_{P}}{GJ}\mathrm{.}$$

These two angles can be used to estimate the projected areas of the leaf to the vertical axis (towards the sun) due to bending and twisting motions, respectively. Here, the projected areas are the product of the cosine of the angles and the original lamina area *A*_*T*_. In order to improve the amount of direct sunlight, a leaf might have the same ratio of projected areas due to bending and twisting^31^. Under this hypothesis, both angles should be equal as

5 $$\frac{{L}_{P}}{E{I}_{X}}(\frac{1}{2}\rho {U}^{2}{L}_{L}{W}_{C}\frac{{L}_{L}}{2})=\frac{{L}_{P}}{GJ}(\frac{1}{2}\rho {U}^{2}{L}_{L}\frac{{W}_{C}^{2}}{4})$$

If we assume that the projected area is large to maximize photosynthesis, then we can write ${A}_{P}^{bend}={A}_{P}^{twist}$ which leads to:

6 $$E{I}_{X}\approx \frac{2{L}_{L}}{{W}_{C}}GJ$$

**Figure 3:**
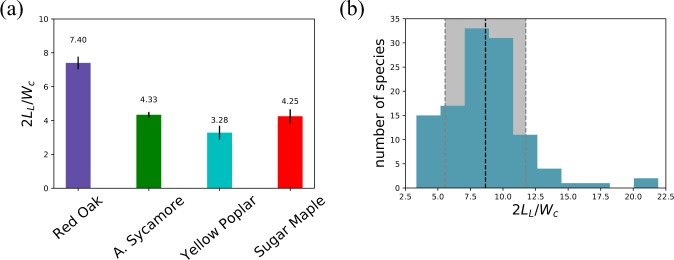
(**a**) Ratio of two times the lamina and the width between the centroids, for the four species tested in the lab. The small error bars indicate that leaves among a species are homothetic, as assumed by an existing model^34^. (**b**) Ratio of two times the lamina and the width between the centroids, for 114 leaves^27^. The complete list of the species and the corresponding value for their ratio is given in Appendix A. The average indicated by the black line is 8.7, and the grey area represents the plus or minus one standard deviation (3.1).

In our case, the ratio 2*L*_*L*_/*W*_*C*_ is found to be in the range 3–8 for species studied in the lab (see Fig. 3(a)). Here, we find that the variation among a species is very small and that each species has a different ratio (Kruskal-Wallis H-test p-value is less than 0.009), meaning that leaves of a single species are both geometrically and statistically similar (in the same shape, but of different sizes).

Moreover, we analyzed 114 more lamina shapes^27^ to extend the number of the data set of 2*L*_*L*_/*W*_*C*_ (Fig. 3(b)). We obtain an average value of 2*L*_*L*_/*W*_*C*_ = 8.7 ± 3.1 which is a bit higher than our lab measurements because of a few outliers (Black Cherry, Corkscrew Willow, Shingle Oak, and White Willow).

**Figure 4:**
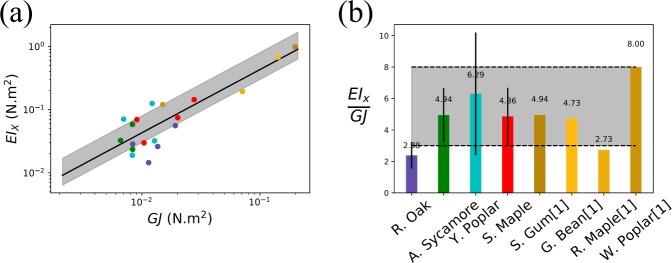
(**a**) Log-log plot of *EI*_*x*_ as a function of *GJ* for 8 different species. The coefficient of linear correlation *r* between *EI*_*X*_ and *GJ* for our data is 0.6, with 15 samples, meaning that we have at least a 93% probability that our data are indeed linearly correlated^32^. The black line is the best fit coefficient 4.3 obtained from only our experimental data - data from the literature^30^ were later added on top. The gray area illustrates the prediction of our model based on the species we studied. (**b**) Ratio of *EI*_*x*_ vs *GJ* for our species and species from Vogel^30^. The gray area indicates the prediction from our model based on the leaf shapes tested in the lab.

To validate our linear relationship of Eq. 6, we plot *EI*_*X*_ as a function of *GJ* as shown in Fig. 4(a). We can see that, as predicted by our model, *EI*_*X*_ linearly increases with *GJ*. The coefficient of linear correlation *r*, quantifying if data are linearly correlated or not, is 0.6, with 15 samples. This result means that we have at least a 93% probability that our data are indeed linearly correlated^32^. The average slope of our lab data is 4.3. This is in agreement with Vogel’s data^30^ which was later on added on the plot. We experimentally find that the ratio *EI*_*X*_/*GJ* of both our data and Vogel’s data^30^ is approximately in a range between 3 and 8. In Fig. 4(b), we plot the ratio *EI*_*X*_/*GJ* for each species and the experimental ratio 2*L*_*L*_/*W*_*C*_ as a gray-shaded region. This plot indicates that these two ratios (*EI*_*X*_/*GJ* & 2*L*_*L*_/*W*_*C*_) are in the same range, showing the validity and robustness of our model.

## Discussion and Conclusion

In this study, we elucidated the relation between the lamina shapes and the petiole mechanical properties through a series of experiments. First, both torsional and tensional tests were performed to evaluate the bending and twisting rigidities of petioles. Second, *μ*-CT scanned images allowed us to estimate the second moment of inertia and cross-sectional area along petioles. Third, both width and length of laminae were measured from images. As a result, we found that among a species the shape of leaves is geometrically similar. Lastly, using mechanical testing performed on leaves in the lab, we showed that it is energetically easier for a leaf to twist than to bend, and furthermore, the famous Vogel’s twist-to-bend ratio is linked to the shape of the laminae. However, it is worth noting the limit of our model: statistically the ratio *EI*_*X*_/*GJ* does not differ between the four different species tested (Kruskal-Wallis H-test gives a p-value of 0.33), preventing us to draw any correlation between the mechanical and geometrical properties of leaves *per species*.

As we described in section 3, twisting mainly occurs in a portion of the petiole close to the lamina, but the twisting length, *L*_*t*_, depends on the species. Mechanically, flexural rigidity and torsional rigidity are composite variables that are influenced both by material and structural properties^33^. However, by just altering geometrical properties, petioles succeeded in changing the location of the twisting area closer to the lamina. With such a geometry, even if the stress is constant throughout the petiole, the strain is smaller close to the stem.

Our simple model is based on wind-induced bending and twisting stress, yet we are not able to capture the detailed dynamics of leaf motions. In particular, we are aware that in nature the bending and twisting of leaves are not static, but more dynamic (e.g. fluttering). While we focused on torsion and traction tests, our approach can also be extended with bending measurements to some extent.

**Figure 5:**
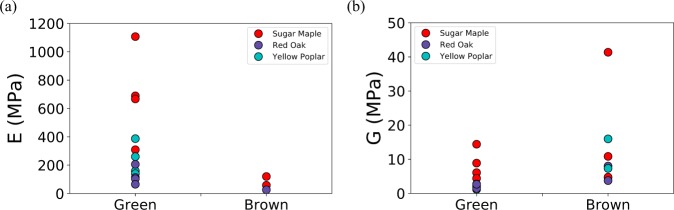
Young’s modulus and twisting modulus obtained on green and brown leaves of three different species. (**a**) The Young’s modulus of brown leaves is drastically lower than the Young’s modulus of green leaves. However, (**b**) the twisting moduli of green and brown leaves are not statistically different.

Our samples were collected during spring/summer when trees grow their leaves and produce food through photosynthesis. However, leaves in the fall might exhibit different mechanical properties as trees are inclined to lose their leaves. We conducted exploratory tests in Fall 2016 on the same trees. We found a drastic decrease in the Young’s modulus (351 ± 303 MPa for green leaves and 68 ± 39 MPa for brown leaves), but not in the twisting modulus (4.1 ± 3.8 MPa for green leaves and 12.1 ± 11.7 MPa for brown leaves), as shown in Fig. 5. However, mechanical testings were difficult to perform, as leaves twisted-off very easily. Therefore, to conclude on this trend, we need more measurements.

## Electronic supplementary material

Supplementary Information
