# Author Correction: How wind drives the correlation between leaf shape and mechanical properties

**DOI:** 10.1038/s41598-020-65139-1

**Published:** 2020-05-12

**Authors:** Jean-François Louf, Logan Nelson, Hosung Kang, Pierre Ntoh Song, Tim Zehnbauer, Sunghwan Jung

**Affiliations:** 1 Department of Biomedical Engineering and Mechanics, Virginia Tech, Blacksburg, VA 24061 USA; 2 Department of Mechanical Engineering, Aix-Marseille Universite, Marseille, France; 3 Department of Biological and Environmental Engineering, Cornell University, Ithaca, NY 14853 USA

Correction to: *Scientific Reports* 10.1038/s41598-018-34588-0, published online 05 November 2018

This article contains an error in Figure 4a, where the x and y axes units are incorrectly given as *GJ*(N.m^2^) and *EI*_X_(N.m^2^) in lieu of *GJ*(mN.m^2^) and *EI*_X_(mN.m^2^), respectively. The correct Figure 4 appears below as Figure 1.

**Figure 1:**
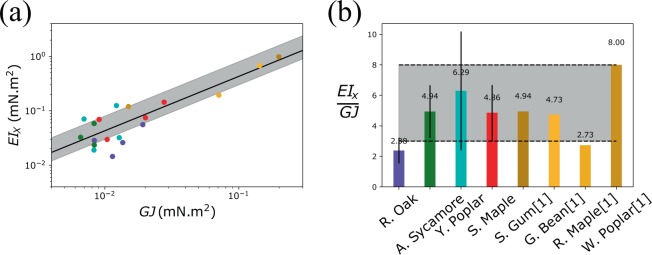
.

