# Supplementary material for: How wind drives the correlation between leaf shape and mechanical properties
Source: Sci Rep. 2018 Nov 5;8:16314. doi: 10.1038/s41598-018-34588-0 (PMC6218545; doi:10.1038/s41598-018-34588-0)
Supplement: Supplementary file 1 — Supplementary Information [file 41598_2018_34588_MOESM1_ESM.pdf]

How wind drives the correlation between leaf shape and mechanical properties

Jean-François Louf[1], Logan Nelson[1], Hosung Kang[1 1], Pierre Ntoh Song[2], Tim Zehnbauer[1], and Sunghwan Jung[1,3]

1 Department of Biomedical Engineering and Mechanics, Virginia Tech, Blacksburg, VA 24061, USA
2 Department of Mechanical Engineering, Aix-Marseille Universite, Marseille, France
3 Department of Biological and Environmental Engineering, Cornell University, Ithaca, NY 48503, USA

Appendix A

Common Name	L_L (cm)	$W_c/2$ (cm)	$2L_L/W_c$
Allegheny Serviceberry	7.02	0.90	7.77
American Beech	8.06	0.87	9.31
American Chestnut	13.87	1.18	11.71
American Hornbeam	8.91	0.88	10.17
American Sycamore	12.95	2.84	4.56
Amur Maple	7.05	0.83	8.52
Asian White Birch	9.48	1.24	7.64
Bigleaf Magnolia	25.43	2.73	9.31
Bigtooth Aspen	9.17	1.49	6.16
Black Cherry	13.17	0.93	14.09
Black Maple	12.35	2.20	5.62
Black Oak	23.15	3.07	7.54
Black Tupelo	14.86	1.42	10.46
Blackthorn	43.16	3.45	12.52
Broadleaf Hawthorn	7.26	1.32	5.50
Callery Pear	6.17	1.36	4.55
Canadian Serviceberry	5.05	0.73	6.93
Carolina Silverbell	12.23	1.45	8.44
Catawba	22.47	3.48	6.46
Cherry Plum	54.60	5.71	9.56
Chestnut Oak	13.28	1.40	9.49
Chinese Elm	4.58	0.43	10.54
Chinese Fringetree	7.29	0.93	7.83
Chokecherry	5.61	0.71	7.89
Common Box	1.97	0.18	11.06
Common Persimmon	11.33	1.17	9.66
Corkscrew Willow	6.16	0.30	20.38
Cornelian Cherry	8.59	1.28	6.71
Cucumbertree Magnolia	14.51	1.52	9.54
Dotted Hawthorn	4.75	0.43	11.03
Downy Birch	4.44	0.62	7.14
Downy Serviceberry	6.61	0.75	8.87
Dutch Elm	5.65	0.82	6.88
Dwarf Hackberry	7.47	0.84	8.92
Eastern Cottonwood	8.63	1.88	4.58
Eastern Redbud	9.91	2.34	4.23
English Elm	7.49	0.79	9.54
English Oak	11.61	1.11	10.46
European Alder	6.39	1.51	4.23
European Beech	6.79	0.85	7.96
European Hornbeam	6.54	0.81	8.12
European Spindletree	5.76	0.54	10.68
European White Birch	4.51	0.53	8.52
Fleshy Hawthorn	4.43	0.61	7.32
Flowering Dogwood	11.46	1.28	8.92
Fragrant Snowbell	19.10	3.80	5.03
Fringe-Tree	10.37	1.07	9.68
Gray Alder	6.41	1.11	5.75

Gray Birch	9.66	1.12	8.60
Green Hawthorn	4.85	0.47	10.32
Hardy Rubbertree	14.84	1.28	11.63
Harlequin Glorybower	16.55	2.38	6.97
Hedge Maple	5.03	1.36	3.71
Higan Cherry	10.57	0.70	15.19
Hophornbeam	9.94	0.86	11.50
Japanese Flowering Crabapple	9.46	0.73	12.95
Japanese Maple	9.53	1.76	5.40
Japanese Snowbell	4.04	0.47	8.63
Japanese Stewartia	7.98	0.87	9.22
Japanese Tree Lilac	10.04	1.08	9.31
Japanese Zelkova	6.86	0.64	10.65
Katsura Tree	6.33	1.17	5.40
Kobus Magnolia	6.13	0.69	8.87
Kousa Dogwood	10.67	1.03	10.40
Lilac	8.04	1.11	7.24
Live Oak	5.59	0.42	13.22
London Plane Tree	11.37	2.18	5.20
Northern Catalpa	25.20	3.22	7.82
Norway Maple	9.67	2.49	3.88
Oneseed Hawthorn	3.19	0.46	6.93
Oriental Cherry	4.30	1.13	3.80
Osage Orange	10.22	0.94	10.92
Paper Birch	5.86	0.90	6.52
Paper Mulberry	14.03	2.07	6.78
Pin Cherry	4.58	0.42	10.88
Pin Oak	11.75	1.50	7.84
Post Oak	19.28	2.23	8.65
Quaking Aspen	11.34	2.25	5.04
Red Maple	8.61	1.73	4.97
Red Oak	17.90	2.55	7.03
River Birch	7.50	1.02	7.32
Roughleaf Dogwood	9.74	1.00	9.70
Sargent Cherry	11.94	1.23	9.72
Scarlet Oak	17.28	1.83	9.44
Scotch Elm	10.71	1.43	7.51
Shingle Oak	10.53	0.66	16.07
Shumard Red Oak	18.13	1.98	9.14
Siberian Crabapple	5.93	0.79	7.48
Siberian Elm	6.03	0.69	8.76
Silver Maple	10.14	2.03	5.00
Slippery Elm	22.77	2.10	10.82
Smooth Hawthorn	2.03	0.33	6.18
Sourwood	15.00	1.39	10.79
Southern Crabapple	6.93	0.98	7.10
Southern Magnolia	16.72	1.37	12.22
Star Magnolia	9.02	0.81	11.12
Striped Maple	15.35	2.78	5.52
Sugar Maple	11.60	2.61	4.44

Swamp White Oak	10.75	1.04	10.35
Sweet Birch	10.18	1.22	8.33
Sweetbay Magnolia	15.91	1.13	14.03
Sycamore Maple	9.30	1.81	5.15
Tea Crabapple	9.91	0.90	11.07
Tuliptree	17.34	3.24	5.35
Umbrella Tree	42.38	4.04	10.48
Virginia Roundleaf Birch	4.06	0.59	6.84
Water Oak	7.71	0.70	11.06
Wayfaringtree	4.89	0.56	8.77
White Mulberry	8.04	1.06	7.60
White Willow	6.16	0.31	20.01
Whitebarked Himalayan Birch	6.12	0.97	6.33
Yellow Birch	14.53	1.70	8.57
Yoshino Cherry	6.88	0.91	7.58
Yulan Magnolia	15.65	2.10	7.45
